# The Impact of the Thai Motorcycle Transition on Road Traffic Injury: Thai Cohort Study Results

**DOI:** 10.1371/journal.pone.0120617

**Published:** 2015-03-31

**Authors:** Janneke Berecki-Gisolf, Vasoontara Yiengprugsawan, Matthew Kelly, Roderick McClure, Sam-ang Seubsman, Adrian Sleigh, Thai Cohort Study Team

**Affiliations:** 1 Monash Injury Research Institute, Monash University, Melbourne, Australia; 2 National Centre for Epidemiology and Population Health, Australian National University, Canberra, Australia; 3 Harvard Injury Control Research Center, Harvard School of Population Health, Boston, Massachusetts, United States of America; 4 School of Human Ecology, Sukhothai Thammathirat Open University, Nonthaburi, Thailand; University of New South Wales, AUSTRALIA

## Abstract

**Objectives:**

The aim of this study was to investigate the impact of motorcycle to car transitioning and urbanisation on traffic injury rates in Thailand.

**Design:**

Analysis of two consecutive surveys of a large national cohort study.

**Setting:**

Thailand.

**Participants:**

The data derived from 57,154 Thai Cohort Study (TCS) participants who provided relevant data on both the 2005 and 2009 surveys.

**Primary and secondary outcome measures:**

Motorcycle and car traffic crash injury self-reported in 2009, with twelve months’ recall.

**Results:**

In 2009, 5608(10%) participants reported a traffic crash injury. Most crashes involved a motorcycle (74%). Car access increased and motorcycle use decreased between 2005 and 2009. Among those who used a motorcycle at both time points, traffic injury incidence was 2.8 times greater compared to those who did not use a motorcycle at either time point. Multivariable logistic regression models were used to test longitudinal and cross sectional factors associated with traffic crash injury: in the adjusted model, cars were negatively and motorcycles positively associated with injury. Living in an urban area was not injury protective in the adjusted model of traffic crash injury.

**Conclusions:**

Ongoing urbanisation in Thailand can be expected to lead to further reductions in road traffic injuries based on transition from motorcycles to cars in urban areas. Cities, however, do not provide an intrinsically safer traffic environment. To accommodate a safe transition to car use in Thailand, traffic infrastructural changes anticipating the growing car density in urban areas is warranted.

## Introduction

Road traffic accidents in Thailand are the third leading cause of death among men; for both sexes aged 15–49 years, road traffic accidents are the second leading cause of death [1]. Of road traffic fatalities in Thailand, 74% are among riders of motorised 2- or 3-wheelers [2]. Although the rate of road traffic deaths has been declining from 2004 onwards, Thailand’s road traffic death rate of 38.1 per 100 000 population remains the highest of South East Asian countries [3]. Arguably, this high road crash death rate is explained by Thailand’s relative position in the developmental transition. Increasing industrialisation, motorisation of the populations within all the ASEAN countries have driven an epidemic of road crash injuries across the region and focused considerable attention on the identification of and implementation of effective solutions.

Because of the predominance of motorcycle injury in the overall road crash injury problem in Thailand, much of the research done to date has been in relation to primary prevention to reduce motorcycle crash risk, and crash helmets as secondary prevention to reduce injury severity in cases where crashes occur [4–6]. The epidemic, however, is not due to the fact that motorcycles have become inherently more risky vehicles, but Thai developmental transition is changing the population’s underlying exposures to road traffic crash risk. To address the problem of motorcycle crash deaths will require more than improving crash risk and helmet wearing (although these will always remain necessary). If development transition has caused the problem, then manipulating the characteristics of this transition may provide the large scale solutions required to curtail the epidemic.

**Fig 1:**
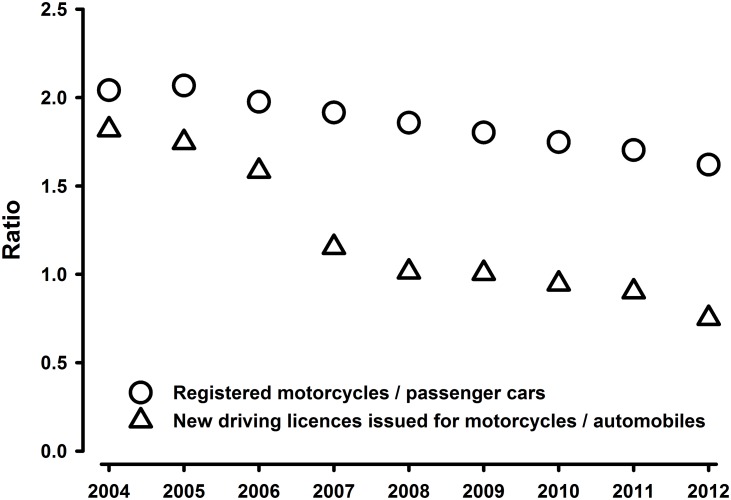
Ratio of registered motorcycles / passenger cars in Thailand between 2004 and 2012; ratio of new driving licences issued for motorcycles / automobiles. Based on source data: Road Transport Thailand—AJTP Information Center [7]

Changing travel mode distribution plausibly provides the greatest opportunity for large scale solutions. Between 2004 and 2012 the overall number of road motor vehicles in Thailand increased from 19.8 to 31.4 million, but during this time period the ratio of registered motorcycles to cars decreased steadily (Fig. 1, based on source [7]). The annual number of driving licences issued for automobiles *increased by 42%* whereas the new driving licences issued for motorcycles *decreased by 41%* during this period (source [7]). Per kilometre driven, car occupants are much less likely to be injured than motorcycle riders. The transition from motorcycle to car use may be responsible for the recent downward trend in Thai road crash deaths.

A second area in which Thailand is undergoing a transition that may underlie the observed changes in the road crash death rate and which may be amenable to intervention is the process of urbanisation. With high density living, road user speeds necessarily reduce and thereby impart a substantial safety benefit. The high density of road user use may provide incentive to maintain or improve quality (and thereby safety) of transport infrastructure. Greater police presence and law enforcement in urban areas may have a positive impact on compliance with road safety laws. Observations that helmet use is much higher in urban than in rural areas [8,9] provides some supports for this suggestion.

A third area of transition are improvements in ambient risk culture and practices attributable to improved health, education and income of individuals, and improved quality and safety of purchased vehicles and built environments. Road traffic mortality is known to increase with initial economic development, followed by a gradual decrease as national income increases [10,11]. Thailand may have reached the latter stage in economic development, characterised by a reduction in road traffic injury and fatality.

To date the relationships between these underlying risk factors and road crash death rates in Thailand have not been explored. It is a principle of the multi-level ecological model of public health that the most effective interventions are those achieved through structural societal change: the identification of the system’s critical structural levers needs to be the focus of global health research [12].

The road crash epidemic in lower and middle income countries is a problem of global health importance, and Thailand’s road injury fatality rate is among the highest in the ASEAN region. Identifying fundamental risk factors amenable to effective intervention is of critical importance. The aim of this study was to quantify the extent to which recent observed reduction in road crashes can be attributed to three key components of the development transition that could potentially be used to curtail the road crash epidemic. This involves quantifying the relationship between individual level changes in transport mode, urbanisation and affluence and connecting these components to changes in injury rates per capita in Thai adults between 2005 and 2009.

## Methods

### Study Design

The data derived from the Thai Cohort Study (TCS), which is an ongoing community-based prospective cohort study of a large sample of adult distance learning Sukhothai Thammathirat Open University (STOU) students residing throughout the country. The TCS is being followed to analyse and understand the overall health-risk transition as Thailand modernises, including the changing incidence of chronic diseases and road traffic injury.

### Population and participants

In 2005 the STOU student register listed about 200,000 names and addresses: a baseline 20-page questionnaire was sent to each student and 87,134 (44%) replied. The 2005 baseline characteristics of cohort participants [13] and comparisons with the population of Thailand [14,15] have been reported previously: the STOU cohort has a slightly higher proportion of females than the general Thai population (54.7% vs. 50.5%); more young adults (51.5% vs. 23.9% were aged between 21 and 30 years) and fewer people aged over 50 (2.0% vs. 24.7%) [14]. Study participants were also less likely to be married and more likely to have completed high school; geographically the main regions in Thailand are well represented in the STOU cohort [14].

Overall the cohort represents well the geo-demographic, ethnic, occupational and socioeconomic status of the young-adult Thai population. This is because most Open University students already have established jobs and because of their work and family responsibilities and modest economic circumstances are unable to leave their locations to attend an on-campus university fulltime. However, they are better educated than the general Thai population and thus are able to respond to complex health questionnaires. In 2009, a follow-up survey was sent and 60,569 (>70%) participants replied: 55% were women and the median age was 34 years (range 19 to 92). Data scanning, verifying, and correcting were conducted using Scandevet, a program developed by a research team from Khon Kaen University. Further data editing was completed using SQL and SPSS software.

Of the 60,569 Thai Cohort Study participants who responded to the survey of 2009, there were 57,154 participants who provided an answer to the question: ‘In the last 12 months how many times did you get injured in a traffic crash’; these participants were included in this study. Study participants who did not respond to the traffic injury question were slightly younger (median 34 vs. 37 years) and more likely to be female (55% vs. 51%) than those who did respond.

### Exposure variables

The exposure variables used in this analysis of transport injuries were reported by Thai Cohort Study participants in surveys undertaken in 2005 and 2009. To address the aims of this study, three main exposure factors were derived from the survey data. First, information regarding study participants’ mode of transport, and 2005–2009 transitions in mode of transport. Second, urbanisation, as determined by participants’ area of residence, and 2005–2009 transitions in area of residence. Third, affluence as measured by household income. Demographic details (age, gender and marital status) were also available from the survey data.

#### Mode of transport

Access to a car or motorcycle was determined from the question ‘which of the following do you, or any member of your household, own? (Bicycle; Car/pick-up/van; Boat; Motorbike; Truck; No vehicle owned). Motorcycle data were further refined by excluding those who selected ‘Don’t use motorcycle’ in response to survey questions about motorcycle helmet use. Exposure is therefore referred to as motorcycle *use* and car *access*. In order to more accurately characterise vehicle exposure over time, longitudinal exposure variables were derived for each of these: access to a car in 2005 as well as 2009 was coded as ‘always’; in 2005 but not in 2009 was ‘stopped’; in 2009 but not in 2005 was ‘started’ and no access in 2005 or 2009 was coded as ‘never’. Similarly, a longitudinal exposure variable was derived for motorcycle use.

#### Urbanisation

Residence in urban or rural areas was determined from responses to the 2005 and 2009 questions: ‘Where is your current permanent home located now? Countryside; City/Town’. A longitudinal variable ‘recent urbanisation’ was created by combining participants’ responses to the 2005 and 2009 questions about area of residence. Those living in rural areas at both time-points were classified ‘country-country’. Those who moved from the country to the city between 2005 and 2009 were classified ‘country-city’. Those who moved from the city to the country were classified ‘city-country’. Those living in urban areas in 2005 and 2009 were classified ‘city-city’.

#### Affluence

Monthly household income was determined from response to the 2009 questions ‘What is your household’s average monthly income (Baht)’; the 9 multiple choice response options were grouped as <7000; 7001–10,000; 10,001–20,000; and ≥20,000 Baht.

#### Demographics

The 2009 TCS survey also included questions about age (date of birth), sex and marital status. Regions in Thailand (Bangkok region/ Central/ North/ North-East/ East/ South) were derived from reported postal codes.

### Outcome variables: Injury

Transport injury was determined from response to the second survey, held in 2009. Participants were asked about the number of times they were injured in a traffic crash in the last twelve months. Study participants were considered to have had a traffic crash injury if they selected ‘one, ‘two’, ‘three’ or ‘four or more’ in response to the question: ‘In the last 12 months how many times did you get injured in a traffic crash’. The number of participants (y) reporting one, two and three injuries (x) fit a power curve ($y=4495.4x^{-2.523}$, R^2^ = 0.998), which indicates that the average number of injuries of those reporting ‘four or more’ can be estimated at 4.29.

For subsequent questions detailing the accident, participants are asked to answer this in relation to their *most serious* traffic injury. The detailed traffic crash information refers to one traffic crash injury only, i.e. the most serious traffic crash injury experienced in the last 12 months before the 2009 survey. Traffic crash injury therefore becomes binary, no longer a count variable.

#### Car crash injury

Study participants were considered to have had a car crash injury if they selected (a) ‘one, ‘two’, ‘three’ or ‘four or more’ in response to the question: ‘In the last 12 months how many times did you get injured in a traffic crash’ and (b) ‘driver’ or ‘passenger’ (but not ‘pedestrian’) in response to the question: ‘When this injury occurred what was your role’ and (c) ‘car/pick-up’ in response to the question: ‘Type of vehicle you were in as driver or passenger?’.

#### Motorcycle crash injury

Study participants were considered to have had a motorcycle crash injury if they selected items (a) and (b) as specified above for car crash injury, but selected (c) ‘motorbike’ in response to the question: ‘Type of vehicle you were in as driver or passenger?’

### Deaths

Road accident fatalities are not included in the road traffic injury analysis. To investigate the possibility of resulting bias in limiting the analysis to non-fatal crashes, an overview of deaths in the cohort is presented. Thai Cohort Study participants have provided their Citizen ID number enabling tracking of deaths in the cohort by matching death records from the Ministry of Interior. The information provided includes sex, date of birth and date of death, description of the cause of death (in Thai), and the International Classification of Disease (ICD-10) code. The most recent death data at the time of this analysis was until March 2010: in total there were 580 deaths among TCS participants. Of these deaths, 83 were due to land transport accidents (ICD codes V01-V89): the first of these occurred in March 2005 and the last in March 2010 (the median date of death was in July 2007). Of the 83 fatal land transport accidents, 14 were due to a motorcycle or three-wheeled motor vehicle accident (ICD V20–39) and 18 were due to a car, pick-up truck or van accident (ICD V40–59). Of the remaining 51 land transport accidents, 39 were due to a pedal bicycle accident (ICD V89.2). Only two of the motorcycle/three-wheeled motor accidents occurred within the approximate 12-month recall period of the 2009 survey (15^th^ June 2008 to 15^th^ June 2009); no car accidents occurred within this time frame. Road traffic fatalities are therefore too few in number to impact the associations reported in this study.

### Analysis

Analyses were performed in *SAS* 9.2 (*SAS* Institute, Cary NC).

To calculate traffic injury incidence per 100 person-years, the sum of reported traffic crash injuries sustained in the last 12 months was divided by the number of study participants, and multiplied by 100. For the total traffic injuries, those reporting multiple injuries contributed more than one injury to the count; ‘4 or more’ was counted as 4.29. Motorcycle injury incidence was calculated by dividing the number of participants reporting a motorcycle injury by the number of motorcycle users in 2009; car injury incidence was calculated by dividing the number of participants reporting a car crash injury by the number of participants with a car owned by self or household member in 2009. Confidence intervals for the incidence rates were calculated by first assuming injury occurrence to have a Poisson distribution, and finding its related confidence interval [16].

Multivariable logistic regression models were used to test the longitudinal and cross sectional factors (mode of transport, urbanisation, affluence, demographic) that were likely to be associated with having at least one traffic crash injury, motorcycle injury, or car crash injury. Interaction effects between the variables of interest (transitions in car access/ motorcycle use and recent urbanisation) were tested but these were not statistically significant at <0.05 in any of the models.

### Ethical considerations

Ethics approval was obtained from Sukhothai Thammathirat Open University Research and Development Institute (protocol 0522/10) and the Australian National University Human Research Ethics Committee (protocols 2004344 and 2009570). Informed written consent was obtained from all participants.

## Results

Of the 57,154 participants who responded to the 2009 survey and provided a response to the question about traffic crash injury, 5608(10%) reported having been injured in a traffic crash in the last twelve months. Of those who reported traffic injuries, 4390 (78%) reported one injury; 834 (15%) reported two, 270 (5%) reported three and 114 (2%) reported four or more injuries.

The most commonly reported vehicle used at the time of the crash was a motorcycle (74%) followed by a car/pickup (12%), bicycle (10%), bus/van/coach (4%) or other (0.5%). The role in the accident was mostly that of driver: 84% of motorcycle crashes, 71% of car/pickup crashes, 89% of bicycle accidents and 50% of other traffic injuries were reported by drivers. Bus/van/coach injuries were mostly reported by passengers (97%).

### 2005–2009 Transitions in motorcycle use and access to a car

Ownership of car/pick-up/van (by self or any member of the household) was reported in 2005 as well as 2009 by 56% of participants (‘always’ access to a car); in 2009 but not in 2005 by 16% (‘started’ access to a car); in 2005 but not in 2009 was reported by 6% (‘stopped’ access to a car); and neither in 2005 or 2009 by 22% of participants (‘never’ access to a car).

Use of a motorcycle was reported in 2005 as well as 2009 by 66% of participants (‘always’); in 2009 but not in 2005 by 8% (‘started’); use in 2005 but not in 2009 was reported by 11% (‘stopped’); and no motorcycle use in 2005 or 2009 was reported by 15% of participants (‘never’). Uptake of a car was therefore more common than cessation of a car, whereas cessation of motorcycle use was more common than uptake of motorcycle use.

### Traffic crash injury in relation to motorcycle use and car access

In 2009, motorcycles were used by 74% of study participants; 72% of participants had access to a car. Table 1 shows how car access and motorcycle use was distributed in the cohort. There are some distinct differences between the patterns of motorcycle vs. car use (Table 1): motorcycles were less common with increasing participant age whereas cars were more common with increasing age. Motorcycle use was most common among those living in the country in 2005 and 2009 whereas car ownership was most common among those living in the city at both time points.

**Table 1:** Distribution of motorcycle use and household car ownership among Thai Cohort Study participants, 2009.

		Study Participants	Motorcycle User	Car Owner*
		N	N (%)^†^	N (%)^†^
All		57154	42198 (74)	41017 (72)
Sex				
	Male	25745	19289 (75)	18960 (74)
	Female	31409	22909 (73)	22057 (70)
Age				
	<25	2543	2032 (80)	1459 (57)
	25–29	13398	10783 (80)	8108 (61)
	30–39	24463	18479 (76)	17728 (72)
	40–49	13066	8844 (67)	10626 (81)
	≥50	3684	2060 (56)	3096 (84)
Marital Status				
	Married	29190	22079 (76)	23738 (81)
	Living together/ de facto	1761	1348 (77)	1163 (66)
	Never married	18193	13028 (72)	10855 (60)
	Separated/Div./Wid.	3498	2452 (70)	2338 (67)
	Missing data	4512	3291 (73)	2923 (65)
Household income				
	= <7000	6203	4853 (78)	2498 (40)
	7001–10000	5599	4526 (81)	2803 (50)
	10001–20000	13520	10952 (81)	8654 (64)
	> = 20000	30308	20919 (69)	26106 (86)
	Missing data	1524	948 (62)	956 (63)
Region				
	BKK	9718	4629 (48)	6733 (69)
	Central	14476	9982 (69)	10221 (71)
	North	10721	9062 (85)	8207 (77)
	Northeast	11321	9444 (83)	8099 (72)
	East	3614	2808 (78)	2731 (76)
	South	7303	6273 (86)	5025 (69)
Recent urbanisation				
	Country-country	20538	17264 (84)	14170 (69)
	Country-city	6543	4859 (74)	4589 (70)
	City-country	3830	3031 (79)	2789 (73)
	City-city	24648	15923 (65)	18349 (74)
	Missing data	1595	1230 (70)	1120 (70)

* Household car owner

^†^ Number and row %

Crude overall traffic injury incidence rates are given in Table 2. Men had higher injury rates than women, and rates declined with increasing age. Injury rates decreased with increasing levels of household income. Rates were highest among those who recently moved from the city to the countryside, and lowest among those living in the city at both time points. Among those who did not have access to a car in 2005 and 2009, traffic injury incidence was 1.7 times greater compared to those who did have access to a car at both time points. Among those who used a motorcycle at both time points, traffic injury incidence was 2.8 times greater compared to those who did not use a motorcycle at either time point.

**Table 2:** Crude traffic injury rates for all traffic injuries, for motorcycle injuries, and car crash injuries.

		Total traffic injuries* ^†^	Traffic injuries /100 person-years*	Motorcycle injuries^†^ ^‡^	Motorcycle injuries / 100 user-years	Car injuries§ ^†^	Car accident injuries / 100 user-years
All		7357	12.9[12.6–13.2]	3470	8.2[8.0–8.5]	539	1.3[1.2–1.4]
Sex							
	Male	3779	14.7[14.2–15.2]	1708	8.5[8.4–9.3]	302	1.6[1.4–1.8]
	Female	3578	11.4[11.0–11.8]	1762	7.7[7.3–8.1]	237	1.1[0.9–1.2]
Age							
	<25	526	20.7[19.0–22.5]	263	12.9[11.4–14.6]	21	1.4[0.9–2.2]
	25–29	2260	16.9[16.2–17.6]	1128	10.5[9.9–11.1]	127	1.6[1.3–1.9]
	30–39	3104	12.7[12.2–13.1]	1461	7.9[7.5–8.3]	236	1.3[1.2–1.5]
	40–49	1176	9.0[8.5–9.5]	509	5.8[5.3–6.3]	120	1.1[0.9–1.4]
	≥50	290	7.9[7.0–8.8]	109	5.3[4.3–6.4]	35	1.1[0.8–1.6]
Marital Status							
	Married	2865	9.8[9.5–10.2]	1406	6.4[6.0–6.7]	277	1.2[1.0–1.3]
	Living together	270	15.3[13.6–17.3]	134	9.9[8.3–11.8]	11	0.9[0.5–1.7]
	Never married	2918	16.0[15.5–16.6]	1373	10.5[10.0–11.1]	158	1.5[1.2–1.7]
	Separated/Div./Wid	558	16.0[14.7–17.3]	244	10.0[8.7–11.3]	43	1.8[1.3–2.5]
	Missing data	745	16.5[15.4–17.8]	313	9.5[8.5–10.6]	50	1.7[1.3–2.3]
Household income							
	= <7000	1224	19.7[18.6–20.9]	549	11.3[10.4–12.3]	25	1.0[0.6–1.5]
	7001–10000	1034	18.5[17.4–19.6]	513	11.3[10.4–12.4]	44	1.6[1.1–2.1]
	10001–20000	1891	14.0[13.4–14.6]	1001	9.1[8.6–9.7]	108	1.2[1.0–1.5]
	> = 20000	3002	9.9[9.6–10.3]	1333	6.4[6.0–6.7]	347	1.3[1.2–1.5]
	Missing data	207	13.6[11.8–15.6]	74	7.8[6.1–9.8]	15	1.6[0.9–2.6]
Region							
	BKK	1091	11.2[10.6–11.9]	379	8.2[7.4–9.1]	67	1.0[0.8–1.3]
	Central	1841	12.7[12.1–13.3]	868	8.7[8.1–9.3]	130	1.3[1.1–1.5]
	North	1421	13.3[12.6–14.0]	683	7.5[7.0–8.1]	122	1.5[1.2–1.8]
	Northeast	1631	14.4[13.7–15.1]	803	8.5[7.9–9.1]	126	1.6[1.3–1.9]
	East	441	12.2[11.1–13.4]	230	8.2[7.2–9.3]	40	1.5[1.0–2.0]
	South	931	12.8[11.9–13.6]	507	8.1[7.4–8.8]	54	1.1[0.8–1.4]
Recent urbanisation							
	Country-country	2814	13.7[13.2–14.2]	1425	8.3[7.8–8.7]	184	1.3[1.1–1.5]
	Country-city	839	12.8[12.0–13.7]	396	8.1[7.4–9.0]	65	1.4[1.1–1.8]
	City-country	550	14.4[13.2–15.6]	259	8.5[7.5–9.7]	47	1.7[1.2–2.2]
	City-city	2943	11.9[11.5–12.4]	1286	8.1[7.6–8.5]	227	1.2[1.1–1.4]
	Missing data	211	13.2[11.5–15.2]	104	9.3[7.6–11.2]	16	1.4[0.8–2.3]
Car ownership 2005/09							
	Always	3271	10.2[9.9–10.6]	1435	6.2[5.9–6.6]		
	Started	1246	14.2[13.4–15.0]	611	8.4[7.7–9.1]		
	Stopped	589	16.7[15.4–18.1]	252	11.9[10.4–13.4]		
	Never	2197	17.7[16.9–18.4]	1151	12.0[11.4–12.8]		
	Missing data	55	13.4[10.1–17.5]	21	8.6[5.3–13.2]		
Motorcycle use 2005/09							
	Always	5686	15.2[14.8–15.6]			355	1.3[1.2–1.5]
	Started	513	11.0[10.1–12.0]			41	1.2[0.9–1.7]
	Stopped	655	10.2[9.4–11.0]			63	1.5[1.2–2.0]
	Never	449	5.4[4.9–5.9]			79	1.2[1.0–1.5]
	Missing	55	13.4[10.1–17.5]			1	0.4[0.0–2.4]

* Where multiple traffic injuries are reported, these are included (one participant can contribute multiple injuries to the injury count). Injury rates are given as number of injuries per 100 person-years [95% confidence interval].

^†^ 12-month recall

^‡^ Motorcycle injuries were only included if the study participant indicated that there was a motorcycle owned by him/herself or a member of the household.

^§^ Car injuries were only included if the study participant indicated that there was a car owned by him/herself or a member of the household.

Motorcycle injury incidence was calculated among participants who had a motorcycle in the household and who used this mode of transport; car crash injury incidence was calculated among those who reported having access to a car (Table 2). Motorcycle injury decreased steeply with increasing age and with increasing level of household income. Among motorcycle users, motorcycle injury rates were highest among those who did not have access to a car in 2005 and 2009. Car crash injury was not clearly related to age or household income, or use of a motorcycle. Car crash injury was higher among males than females.

Multivariate modelling of traffic injury, motorcycle injury and car accident injury is shown in Table 3. Not having access to a car, particularly ceasing to have access to a car between 2005 and 2009, was associated with transport injury. Not having access to a motorcycle, particularly not having access to a motorcycle at both surveys, was negatively associated with injury. Increasing levels of household income was mildly, but statistically significantly, associated with injury. In the fully adjusted model taking motorcycle use and car access into account, living in the city was associated with traffic crash injury.

**Table 3:** Fully adjusted models of any traffic injury, motorcycle (m.c.) injury, and car crash injury.

		Any traffic injury among all participants		Motorcycle injury among m.c. users		Car accident injury among car owners	
		4811/50067*		3012/37216*		466/36396*	
		OR [95%CI]	p-value	OR [95%CI]	p-value	OR [95%CI]	p-value
Sex							
	Male	1.3[1.3–1.4]	<0.0001	1.3[1.2–1.4]	<0.0001	1.6[1.3–2.0]	<0.0001
	Female	1 [REF]		1 [REF]		1 [REF]	
Age							
	<25	1.4[1.3–1.7]	<0.0001	1.6[1.4–1.9]	<0.0001	1.2[0.7–1.9]	0.56
	25–29	1.2[1.1–1.3]	<0.0001	1.2[1.1–1.4]	<0.0001	1.3[1.0–1.6]	0.05
	30–39	1 [REF]		1 [REF]		1 [REF]	
	40–49	0.8[0.8–0.9]	0.0001	0.8[0.7–0.9]	<0.0001	0.7[0.6–0.9]	0.01
	≥50	0.8[0.7–1.0]	0.02	0.7[0.6–0.9]	0.001	0.7[0.5–1.0]	0.08
Marital Status							
	Married	1 [REF]		1 [REF]		1 [REF]	
	Living together/ de facto	1.2[1.1–1.5]	0.008	1.2[1.0–1.5]	0.03	0.8[0.4–1.4]	0.41
	Never married	1.4[1.3–1.4]	<0.0001	1.3[1.2–1.4]	<0.0001	1.2[1.0–1.5]	0.09
	Separated/Div./Wid.	1.7[1.5–1.9]	<0.0001	1.6[1.4–1.9]	<0.0001	1.8[1.3–2.5]	0.001
Household income							
	= <7000	1.2[1.1–1.3]	0.0002	1.1[1.0–1.2]	0.24	0.7[0.4–1.1]	0.15
	7001–10000	1.2[1.1–1.4]	0.0001	1.2[1.0–1.3]	0.02	1.1[0.8–1.7]	0.47
	10001–20000	1 [REF]		1 [REF]		1 [REF]	
	> = 20000	0.9[0.8–0.9]	0.001	0.8[0.8–0.9]	0.0003	1.2[0.9–1.5]	0.21
Region							
	BKK	1 [REF]		1 [REF]		1 [REF]	
	Central	1.0[0.9–1.1]	0.93	1.1[0.9–1.2]	0.33	1.3[1.0–1.9]	0.08
	North	0.9[0.8–1.0]	0.16	1.0[0.8–1.1]	0.76	1.7[1.2–2.4]	0.004
	Northeast	1.0[0.9–1.2]	0.57	1.1[1.0–1.3]	0.19	1.8[1.2–2.5]	0.002
	East	0.9[0.8–1.1]	0.22	1.0[0.9–1.2]	0.78	1.4[0.9–2.3]	0.12
	South	0.9[0.8–1.0]	0.06	1.0[0.9–1.2]	0.97	1.0[0.7–1.6]	0.84
Recent urbanisation 2005/09							
	Country-country	1 [REF]		1 [REF]		1 [REF]	
	Country-city	1.0[0.9–1.1]	0.70	1.0[0.9–1.1]	0.78	1.1[0.8–1.5]	0.63
	City-country	1.1[1.0–1.3]	0.02	1.1[1.0–1.3]	0.19	1.2[0.8–1.7]	0.30
	City-city	1.2[1.1–1.3]	<0.0001	1.1[1.0–1.2]	0.02	1.1[0.8–1.3]	0.66
Car ownership 2005/09							
	Always	1 [REF]		1 [REF]			
	Started	1.2[1.1–1.3]	0.002	1.2[1.1–1.3]	0.002		
	Stopped	1.5[1.3–1.7]	<0.0001	1.6[1.4–1.9]	<0.0001		
	Never	1.3[1.2–1.4]	<0.0001	1.6[1.4–1.7]	<0.0001		
Motorcycle ownership 2005/09							
	Always	1 [REF]				1 [REF]	
	Started	0.7[0.7–0.8]	<0.0001			1.0[0.7–1.4]	0.99
	Stopped	0.6[0.5–0.7]	<0.0001			1.2[0.9–1.6]	0.20
	Never	0.3[0.3–0.4]	<0.0001			1.1[0.8–1.5]	0.41

* Number of study participants with injury / number of study participants exposed.

The model of motorcycle injury was very similar to the general traffic injury model (because the majority of transport injuries were motorcycle injuries). Male gender, young age, not being married, lower levels of household income and not having access to a car are associated with motorcycle injury among motorcycle users. Among those with access to a car, male gender, age 25–29, being separated or divorced, and living in the North or Northeast region of Thailand were associated with car crash injuries. Household income was not associated with car accident injury among car owners. Urbanisation was not statistically significantly associated with motorcycle injury or car injury in the fully adjusted models.

## Discussion

Motorcycles were more commonly used by participants living in the country and cars were most common among urban dwellers. Traffic crash injury rates were lower among urban and recently urbanised Thai Cohort Study participants than among those living in the countryside, but this difference was fully explained by the uptake of cars and cessation of motorcycle use by participants living in urban areas. Urbanisation was not an independent predictor of traffic injury. Household income was associated with car ownership; after adjusting for car and motorcycle ownership, household income was not a strong predictor of traffic injury.

Although traffic injury rates can be expected to decrease further with the ongoing transition from motorcycle to car use, urbanisation itself does not reduce traffic crash injury. Out of the five key risk factors for road traffic injury: speed, drink-driving, motorcycle helmet use, seatbelts and child restraints, Thailand has set a measurable target for only one: motorcycle helmet use[3]. To accommodate a safe transition to car use, accident preventative measures, particularly measures anticipating the increased use of cars in urban areas, are warranted.

Pedestrian fatalities account for 34% of road user deaths in low-income countries and 11% in middle income countries of South-East Asia [3]. In low income countries world-wide, 45% of road traffic deaths are among pedestrians. The situation in Thailand, a middle income country, is quite different: pedestrian fatalities account for 8% of road traffic deaths, but 2–3 wheelers, which are also classified as vulnerable road users, account for 74%. Increasing motorisation in countries undergoing economic transition usually leads to an initial increase of pedestrian fatalities until improvements in the traffic infrastructure have been made (such as the separation of pedestrians from motorised vehicles) and fatalities gradually decrease [11]. In Thailand, the increase in car traffic will have to be accommodated with traffic infrastructure adaptations that protect the predominant vulnerable road user group in Thailand: motorcycle riders.

This study has limitations. The main limitation is the lack of motor vehicle use quantification such as an approximation of the kilometres travelled per year per participant. In particular, quantification of car use is lacking: only car ownership by self or household was asked in the surveys; usage was not. Future studies measuring the impact of urbanisation on distance travelled per vehicle type and traffic injury could fill the gaps in knowledge.

The accuracy of the findings of this study could be affected by traffic crashes that resulted in loss of the vehicle, where the vehicle was not replaced after the crash. The effect of this would be reflected in a high number of traffic crash injuries among those who ‘stopped’ having access to a car and those who stopped using a motorcycle between 2005 and 2009. As this was not the case (Table 2), it is likely that crashes resulting in total loss of vehicle without replacement were uncommon.

This analysis is limited to transitions in motorcycle and car use but did not consider transitions in public transport use or non-motorised transport use as predictors of traffic injury. Particularly in a more detailed study of transport use quantification, trends in non-motorised transport and public transport can be taken into account: ongoing urbanisation is increasing the need for public transport from outer suburbs of Bangkok to the city [17]. How this affects transport injury in Thailand remains to be investigated.

And finally, the generablisability of the results of this study is limited by the representativeness of the TCS sample. Although the TCS is representative of the socioeconomic status of the young-adult Thai population, older Thais are underrepresented. Furthermore, because all TCS participants enrolled in further education, this cohort can be considered an aspirational cohort. In some aspects, trends observed among TCS participants could precede trends in the general population: for example, the uptake of cars may be observed among aspirational young adult Thais in advance of the general population. The trends identified in this study will need to be confirmed with ongoing monitoring of car and motorcycle registrations and road traffic crash reports.

### Conclusions

Traffic injury rates among Thai Cohort Study participants were increased by motorcycle use and reduced by car use. The urban-rural difference in traffic crash injury rates can be fully attributed to urban-rural differences in car and motorcycle use, with motorcycles more common in rural areas and cars more common in urban settings. Ongoing urbanisation in Thailand can therefore be expected to lead to further reductions in road traffic injuries based on ongoing transition from motorcycles to cars. Cities, however, do not provide an intrinsically safer traffic environment. There is a need for infrastructural changes to accommodate the increasing car density in urban areas. Particularly the separation of cars from vulnerable road users (predominantly motorcycles in Thailand) is warranted to prevent the increased car density from leading to reversal of the downward road fatality trend.
